# Progress towards a targeted biorefinery of *Chromochloris zofingiensis*: a review

**DOI:** 10.1007/s13399-022-02955-7

**Published:** 2022-06-27

**Authors:** Eleanor E. Wood, Michael E. Ross, Sébastien Jubeau, Valéria Montalescot, Michele S. Stanley

**Affiliations:** 1 University of the Highlands and Islands (UHI); Scottish Association for Marine Science (SAMS), Scottish Marine Institute, Oban, PA37 1QA UK; 2 Xanthella Ltd, Malin House, European Marine Science Park, Dunstaffnage, Argyll, Oban PA37 1SZ Scotland, UK; 3 Cargill, 1 rue de Sèves, Baupte, France

**Keywords:** Biorefinery, *Chromochloris zofingiensis*, Astaxanthin, Lipids, Microalgae, Downstream processing

## Abstract

Biorefinery approaches offer the potential to improve the economics of the microalgae industry by producing multiple products from a single source of biomass. *Chromochloris zofingiensis* shows great promise for biorefinery due to high biomass productivity and a diverse range of products including secondary carotenoids, predominantly astaxanthin; lipids such as TAGs; carbohydrates including starch; and proteins and essential amino acids. Whilst this species has been demonstrated to accumulate multiple products, the development of an integrated downstream process to obtain these is lacking. The objective of this review paper is to assess the research that has taken place and to identify the steps that must be taken to establish a biorefinery approach for *C. zofingiensis*. In particular, the reasons why *C. zofingiensis* is a promising species to target for biorefinery are discussed in terms of cellular structure, potential products, and means to accumulate desirable components via the alteration of culture conditions. Future advances and the challenges that lie ahead for successful biorefinery of this species are also reviewed along with potential solutions to address them.

**Supplementary Information:**

The online version contains supplementary material available at 10.1007/s13399-022-02955-7.

## Introduction 

A biorefinery is an integrated facility where downstream processes are combined to produce at least three products from biomass [1, 2]. Generally, the cost of algal cultivation for a single product can be prohibitively high. Separation of multiple products has the potential to increase the value of the biomass, whilst concomitantly promoting sustainable processing, reducing waste, and contributing towards meeting the world’s rising demand for food, water, and energy [1, 2]. By identifying and utilising multiple compounds within the biomass, with priority given to high-value products, the process of cultivation should become more economically viable [3, 4]. The most economic methods and unit operations must be used because increased processing is required. This can often have multiple benefits; for example, reduced energy input can improve the operational costs and environmental sustainability of the process [5]. Three key examples of sequential biorefinery processes of microalgae from research papers are as follows: the separation of polysaccharides, proteins, and phycoerythrin from *Porphyridium* species [6]; the separation of polysaccharides, proteins, and antioxidants from *Chlorogloeopsis fritschii* [7]; and separation of starch, pigments, proteins, and sugars from *Tetraselmis suecica* [8]. Such processes are yet to be demonstrated at a commercial scale.

The species used for algal biorefinery should be selected based on the high-value molecules that can be produced and used for multiple applications so that the production processes can be economically viable. In this review paper, the potential for the use of *Chromochloris zofingiensis* as a species for biorefinery will be discussed. The implementation of a biorefinery approach could result in successful commercialisation of this species. From an industrial perspective, it is important to know the species and strain being cultured so barcoding of strains should take place regularly. *Chromochloris zofingiensis* formerly had six other names: *Chlorella zofingiensis*, *Bracteacoccus cinnabarinus*, *Muriella zofingiensis*, *Mychonastes zofingiensi*s, *Chromochloris cinnabarina*, and *Bracteacoccus minutus*. There are currently 22 strains available from culture collections globally, 12 of which have had the name updated to *Chromochloris zofingiensis* (Online Resource 1). The original holotype, Culture Collection of Algae at Göttingen University (SAG 211–14), has been shared across several culture collections including the American Type Culture Collection (ATCC 30,412), Culture Collection of Algae and Protozoa (CCAP 211/14), Culture Collection of Algae at The University of Texas at Austin (UTEX 32), Culture Collection of Algae of Charles University Prague (CAUP H6503), Microbial Culture Collection at the National Institute for Environmental Studies (NIES 2175), and Collection of Algae of Leningrad University (CALU 190). Of these, ATCC 30,412, SAG 211–14, and UTEX 32 are the most cited in the literature. As well as the strains listed in Online Resource 1, there are several others that have been cited but cannot be tracked down to any culture collection database, putatively due to researchers maintaining private cultures or their loss from public collections. Within this review paper, the species will be referred to according to the strain number, if available, to make comparisons between different isolates, or *C. zofingiensis*.

*C. zofingiensis* can grow phototrophically, mixotrophically, and heterotrophically and its high-density growth, scalability, and tolerance to a wide range of environmental conditions make it a desirable species for biotechnology [9–13]. The different growth modes can be used to tailor the biochemical composition of the cells most commonly to increase the biomass production and concentrations of secondary carotenoids and lipids, turning the cells from green to red. This can be achieved through applying stress-inducing conditions to phototrophic cultures, or through mixotrophic or heterotrophic growth modes. Different molecules accumulate across the growth cycle, in accordance with metabolism and the nutrients available in the media, specifically carbon and nitrogen of which there is a direct inverse relationship. Therefore, when aiming to achieve carotenoid accumulation the C:N should be high, for example, 200:1 [14]. The cultivation strategy (batch, fed-batch, or continuous) also influences the biomass concentration and composition of the algae.

Suggested products from biorefinery of *C. zofingiensis* include the carotenoids astaxanthin, canthaxanthin, β-carotene, and lutein, lipids such as triacylglycerides (TAGs), and carbohydrates including starch, as well as proteins or amino acids [2, 5, 9, 15–17]. At the current market price and demand, arguably the most economical approach would be to target the production of astaxanthin from *C. zofingiensis*, since it carries a high value and already has an established and unfulfilled market for nutraceuticals, aquaculture, and cosmetics [18–21]. A notable feature of *C. zofingiensis* is that it can produce astaxanthin and TAGs simultaneously [9, 22]. Even so, obtaining three or more products could create higher revenue than using the whole biomass or extracting an astaxanthin-lipid product only. TAGs can be used for biodiesel or ingested as nutraceuticals [23, 24]. Starch can be used as a bioethanol feedstock or for bioplastics [25, 26]. Other components, including proteins and other carbohydrates, are less explored for this species but could be utilised as by-products for feed, fertilisers, biostimulants, enzymes, and cosmetics. The availability of multiple products allows production to be more resilient to changes in market value and consumer demand.

Natural astaxanthin production from *C. zofingiensis* could compete with *Haematococcus pluvialis* in the commercial market [17]. Not only does *H. pluvialis* have a lower growth rate than *C. zofingiensis* but it is also frequently contaminated by *Paraphysoderma sedebokerense*, a destructive fungus that can cause major losses of cell culture and hence astaxanthin productivity [27]. *C. zofingiensis* has also been shown to be susceptible to this fungus although *P. sedebokerense* has demonstrable preference towards infecting *H. pluvialis* [28]. This problem demonstrates the need for diversification of microalgal species to build resilience in the wider industry which applies when producing any commodity industrially.

*C. zofingiensis* is one of the most commonly mentioned microalgal species for implementation into a biorefinery [5, 9, 16, 17, 29]. However, despite being used as an example, limited experimental research has taken place to obtain multiple products. The aim of this article is to review the current research on *C. zofingiensis*, identify areas that have not been explored, and lay the foundations for successful biorefinery of this species. The main objectives of this article are to (i) review cell structure information to aid selection of cell disruption techniques, (ii) consolidate the potential products that can be produced by this species, (iii) highlight optimal culture conditions for accumulation of specific products, (iv) summarise the research on the stages of biorefinery that have been investigated, and (v) identify the challenges that are preventing successful biorefinery of this species and recommend solutions for these.

## Variation of cellular structure in *C. zofingiensis*

The cellular structure of *C. zofingiensis* and any microalgal species varies depending on the culture conditions. It is important to know the structure of the cells being processed because when establishing a biorefinery this will influence the downstream processes that are used.

### Cell size, outer morphology, and reproduction

*C. zofingiensis* is a unicellular, non-motile, freshwater Chlorophyte [9, 30]. *C. zofingiensis* cells are spherical or oval in shape [31]. The cell size is 2–17 µm which varies depending upon the life stage of cell growth [9, 32]. Azaman et al. [33] showed that the average cell size of ATCC 30,412 increased under mixotrophic conditions from 4 to 6–9 µm, and the range increased from 1–11 to 3–15 µm due to the accumulation of lipids and starch.

**Fig. 1:**
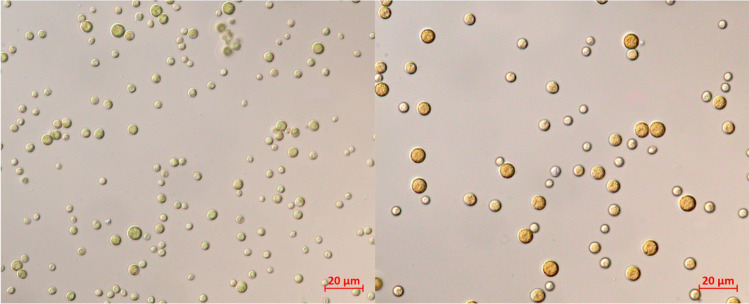
SAG 211/14 *C. zofingiensis* cells under phototrophic (left) and mixotrophic (right) conditions at × 63 magnification taken using a Zeiss Axio Imager.A2 light microscope

Reproduction is asexual, with 4–64 autospores forming from parental cells to create daughter cells in a three-stage process of growth, ripening, and division [9, 32]. *C. zofingiensis* divides by a consecutive pattern of multiple fissions and synchronises growth and cell division according to illumination [34]. Chen et al. [14] showed that nitrogen deprivation and high light caused the parental cells, which turned red with carotenoids, to release more autospores because the percentage of small cells increased from 22 to 46% of the culture. The identification of the shape and size of cells under different conditions could assist in selection of cells with an ideal cell structure and composition for biorefinery (Fig. 1). Certain morphotypes may be more amenable to cell disruption hence, different strategies could be employed during downstream processing.

### Cell wall

It is important to recognise the structure of the cell wall to implement the most effective cell disruption technique for developing future biorefinery strategies. *C. zofingiensis* has a smooth cell wall with an irregular network of ribs [31, 32, 35, 36]. Secondary carotenoids may function as a substrate for the synthesis of sporopollenin and assist in the development of the outer, trilaminar cell wall in Chlorophyceae [37, 38]. This implies that the cell wall becomes more difficult to break as carotenoids accumulate. The ATCC 30,412 cell wall contains mucilage, which can be seen from SEM images [36]. Such mucilage could be exopolysaccharides (EPS) which were identified by Zhang et al. [39]. There is some discrepancy about the composition and thickness of the cell wall which needs clarification in relation to culture conditions to implement an effective cell disruption stage in the biorefinery process [31, 37, 40–44].

### Locations of molecules within the cell

The proportion of organelles within *C. zofingiensis* varies depending on the culture conditions and the stage of growth. The chloroplast is located peripherally in the cytoplasm and has been reported to occupy 12–50% of the cell volume [9, 45]. In terms of biorefinery, the chloroplast could be of potential value, since cells could be mildly disrupted thereby leaving the chloroplasts intact, facilitating the recovery of membrane-bound pigments or starch granules. Young *C. zofingiensis* cells have one chloroplast or 1–4 thin parietal chloroplasts abundant with small starch granules which could be an easily separated product [9, 31, 36, 41]. Starch granules, within the chloroplast, increased nearly fivefold in heterotrophic cells, representing 30.9% of the chloroplast volume and 10.2% of the overall cell [45]. Mature cells contain multiple chloroplasts because the chloroplast cell membranes become thicker and break into numerous smaller fragments [31, 41]. Therefore, if aiming to obtain whole chloroplasts, disrupting mature cells mildly could be preferable. Nevertheless, observations of chloroplast degradation and absence have been reported in ATCC 30,412 cells grown heterotrophically or mixotrophically [20, 46]. Understanding changes in the chloroplasts under different conditions for different strains could allow targeted cell disruption and fractionation of cellular products in a biorefinery process.

Cellular components can exist in different forms and locations depending on the culture conditions. Astaxanthin accumulates outside of the chloroplast, as it is not coupled to the photosynthetic apparatus, in cytoplasmic lipid droplets where it may prevent peroxidation of fatty acids due to the export from the plastid to the cytoplasm [2, 11, 47]. This means that astaxanthin and lipids will extract together when cells are broken. Lipid bodies are usually observed peripherally but, under stressed conditions, lipid bodies exist exclusively in the cytosol and merge together to form a layer surrounding the shrunken chloroplast which has decreased starch levels [9, 48]. Another observation is that lipid bodies and starch accumulate in the middle of the cells under mixotrophic conditions [33]. Zhang et al. [20] showed that glucose increased the astaxanthin accumulation within the first 24 h, but this was hindered after 48 h due to a lack of biosynthesis sites in the chloroplasts. Similarly, Zhang et al. [46] found that ATCC 30,412 cells were smaller with fewer lipid droplets and starch granules 48 h after glucose depletion, and it was proposed that such storage molecules are degraded to provide carbon and energy. However, large lipid droplets (up to 600 nm in length) found in nitrogen deprived with glucose ATCC 30,412 cultures and nitrogen-deprived UTEX 32 cultures may be preferable for ease of separation of products [20, 49]. Knowing the location and size of molecules within the cell at different growth phases can assist in tailoring harvest time, cell disruption, and product separation techniques for biorefinery.

## Products from *Chromochloris zofingiensis*

**Fig. 2:**
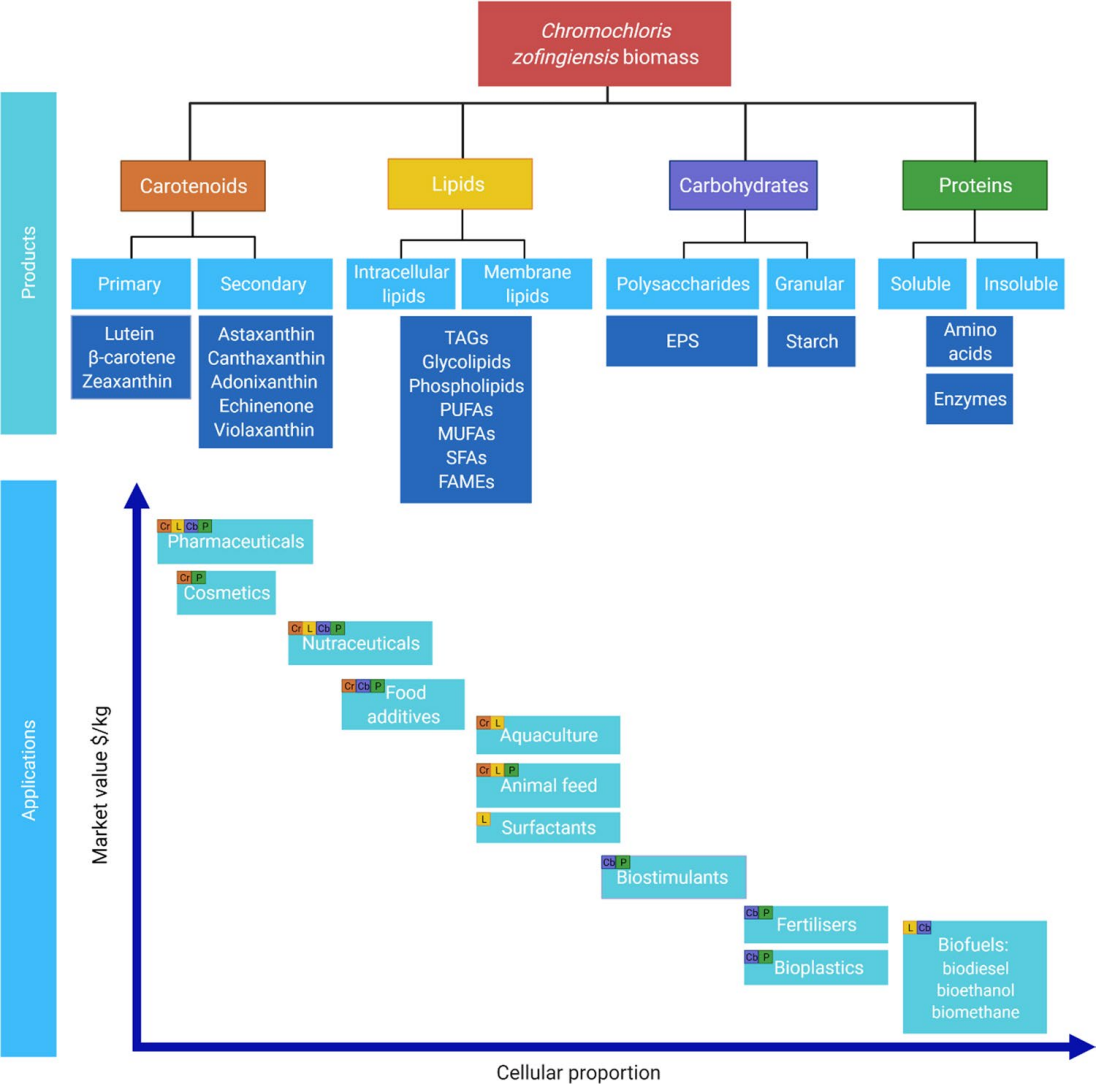
Potential products and applications of extracted components from *C. zofingiensis.* The coloured squares represent the products that can be used for each application

Biochemical analysis of *C. zofingiensis* has revealed an abundance of potential products (Fig. 2). Although many molecules carry value, their respective order of separation from the biomass should depend upon their concentration, properties, market value, and demand. The components that exist in lower concentrations tend to carry more value, e.g., astaxanthin, and those that exist as a larger proportion of the cell are usually less valuable, e.g., lipids. Here, the most promising products that could be obtained through biorefinery of this species are discussed namely, carotenoids > lipids > carbohydrates > proteins (Table 1). It is challenging to compare published data due to variations in methods used by different research groups as well as different styles of reporting data. Additionally, regulations vary in different countries and geographical areas meaning that products from microalgae that are approved in one location may require further development elsewhere [50].

**Table 1:** Key products from *C. zofingiensis*, applications, market value, and maximum quantities reported in the literature

Component		Application	Market value	Maximum quantities in *C. zofingiensis* reported in the literature	References
Carotenoids		Antioxidants, colourants, food, feed, cosmetics, and nutraceutical properties	$2.0 billion by 2026	~ 10 mg/g	[17, 50, 51]
	Astaxanthin	Anti-inflammatory, anti-cancer, treatment of cardiovascular diseases, pigmenter for aquaculture, antioxidant, immunomodulation, anti-bacterial, pharmaceuticals, nutraceuticals, cosmetics, food, feed, improves brain function	$2000-$15,000/kg currently ~ 15t of pure astaxanthin is produced globally so total market value is currently $30–225 million Projected to be $770 million from microalgae by 2024	0.27% DW 194.5 mg/L astaxanthin	[9, 21, 23, 51–61]
	Canthaxanthin	Creates tan colour, antioxidant, pigmenter in aquaculture, poultry, and food, anti-cancer	Projected to be $220.59 million by 2019 globally, $90.79 million of this was in Europe $429/kg	30.6% of total carotenoids	[21, 51, 54, 62–64]
	β-Carotene	Food colourant, antioxidant, anti-cancer, prevents night blindness, prevents liver fibrosis, pigmenter for aquaculture, cosmetics, nutraceuticals, feed	Projected at $344.22 million in 2019 globally, $140.89 million of this was in Europe $227/kg	7.18 mg/g or 34.64 mg/L mutant 4.1 mg/L phototrophic 0.3 mg/g heterotrophic	[15, 21, 51, 54, 65–69]
	Lutein	Prevents cataract and age-related macular degeneration, antioxidant, anti-cancer, prevents cardiovascular diseases, food additive E161b, yellow colouration of egg yolk (feed additive), pigmentation of animal tissues, pharmaceuticals, cosmetics, food colourants, feed supplements, and cosmetics	$308 million in 2018 Projected as $269.07 million in 2019 globally, $113.74 million of this was in Europe $45–1451/kg	8.74 mg/L mixotrophically or 1.28 mg/g phototrophically	[21, 51, 52, 64, 65, 70–72]
Lipids		Pharmaceuticals, nutraceuticals, food technology, energy creation, biodiesel	For biodiesel $4.93/L PBR, $2.97/L open pond	65.10%	[16, 71, 73]
	TAGs	Biodiesel, nutraceuticals	$600.6 million in 2017 (medium chain) $809.3 million by 2022 (medium chain) $1.5/L	89.1% of TFAs 85.5% of total lipids 40% DW and 243 mg/L/day	[46, 74–76]
	Glycolipids and phospholipids	Cosmetics, personal care products, surfactants, functional food ingredients, pharmaceuticals such as anti-inflammatories, drug delivery	Glycolipids €4.5/kg	19.1% of total lipids combined Glycolipids 12.95% DW, 50.7% of total lipids Phospholipids 1.92% DW, 7.5% of total lipids	[77–83]
	PUFAs	Nutraceuticals, feed additives, aquaculture, pharmaceuticals, anti-inflammatory, prevents type 2 diabetes, obesity, cardiovascular and other metabolic diseases	$82 million $30–80/kg EPA and DHA $4 billion by 2022	EPA 0.58% and DHA 0.74% of TFAs	[5, 60, 84–86]
Carbohydrates		Biofuels, biostimulants, bioplastics, fertilisers, pharmaceuticals, nutraceuticals, food additives, cosmetics	$0.5/kg	66.90%	[25, 52, 71, 87, 88]
	Starch	Bioplastics, biofuel, bioethanol	€2 to €4.5/kg	43% DW	[25, 26, 85, 88, 89]
	EPS	Antioxidant, anti-tumor, stabilising, emulsifying, viscosifying, antibiofilm, bioflocculant, food additives, pharmaceuticals, nutraceuticals, cosmetics	$1.1 to $1000/kg	208.4 mg/L	[39, 52, 60, 64]
Proteins		Food and feed additives, thickening, foaming, gelling and emulsifying agents, nutraceuticals, biostimulants, pharmaceuticals, anti-inflammatory, anti-cancer, cosmetics, antioxidants, photoprotective properties, bioplastics	$0.5 to £40/kg	33.15% DW	[52, 60, 83, 87, 90]
	Amino acids	Human nutrition, supplements, vaccines, therapeutic anti-bodies, fertilisers, food technology, biostimulants	n/a	n/a	[71, 87]

### Carotenoids

In *C. zofingiensis*, the primary carotenoids; the structural and functional components of photosynthesis, are β-carotene, lutein, and zeaxanthin which are present inside the chloroplast. The secondary carotenoids are astaxanthin, canthaxanthin, adonixanthin, echinenone, violaxanthin, and ketolutein which accumulate in the lipid droplets outside of the chloroplast [9, 10, 14, 15, 62, 91]. Secondary carotenoids are considered to be more powerful antioxidants than primary carotenoids as they accumulate under ‘stress’ conditions [9, 72]. Secondary carotenoids are presumed to protect chlorophyll and cellular components, serve as antioxidants preventing accumulation of oxygen radicals, and/or act as a hydrophobic layer to reduce osmotic stress [92–94].

Under high light intensity and nutrient deprivation, the major pigments have been demonstrated to be astaxanthin and its precursor canthaxanthin [37, 50, 62]. There is some variation depending on the culture conditions; for example, Minyuk et al. [62] showed that astaxanthin ranged from 43.7 to 46.5% and canthaxanthin ranged from 25.5 to 30.6% of the total carotenoids when comparisons were made between phototrophic and mixotrophic cultures. The most promising carotenoids produced by *C. zofingiensis* are astaxanthin, canthaxanthin, β-carotene, and lutein because each carries a high value as an individual purified product, has an already established market, and exists at relatively high levels within the cells (Table 1). Canthaxanthin is FDA approved for use as a colour additive in foods, lutein is approved in the EU as E161b, and β-carotene is FDA approved as a nutrient supplement to be added in infant formula which can be digested and transformed to vitamin A, a colour additive for food products, drugs (with the label of “only as a colour additive”), and cosmetics. There may also be value as a group of carotenoids. From 2016 to 2021, the carotenoids market has been led by astaxanthin followed by β-carotene and lutein [51]. The carotenoid market is expected to grow from $1.5 billion in 2017 to $2.0 billion by 2026, representing a CAGR of 5.7% [51].

#### Astaxanthin

Astaxanthin is the principal target compound for *C. zofingiensis* due to its high value and demand. It functions as an internal sunscreen and antioxidant by absorbing excess light and quenching reactive oxygen species (ROS). Currently, the astaxanthin market is dominated (95%) by synthetic production, with chemicals from the petrochemical industry. This is because the production cost of naturally derived astaxanthin from *H. pluvialis* is prohibitively high at $2500–7000/kg compared to $1000/kg synthetically [59]. Nevertheless, natural astaxanthin is superior in terms of antioxidant activity and largely exists in the preferable esterified form compared to synthetic sources [9, 95]. Natural astaxanthin is predominantly esterified with fatty acids, in the form of either monoesters or diesters [96]. Interestingly, ATCC 30,412 was observed to contain a higher percentage of astaxanthin diesters (76.3% of the total astaxanthin) than monoesters (18% of total astaxanthin) compared with *H. pluvialis* (35.5% of total astaxanthin as diesters and 60.9% as monoesters) [97]. This difference could be beneficial as diesters are more stable during storage than monoesters [98]. At present, *H. pluvialis* is the only natural source of astaxanthin that has been approved by the Food and Drug Administration (FDA) for human nutrition; other sources such as yeast and bacterial species have only been approved for aquaculture [99]. Therefore, astaxanthin from alternative species of algae, including *C. zofingiensis*, would require legislative approval. *H. pluvialis* can accumulate astaxanthin at 5–7% DW (Algalif.is) or 35 mg/L [100]. In comparison, the highest reported dry weight quantity in *C. zofingiensis* is much lower on a cellular basis at 0.27% DW [61], but is higher in terms of concentration per volume, 194.5 mg/L astaxanthin [61]. This is due to the high growth rates and cell densities of *C. zofingiensis* for example, 73.7 g/L heterotrophic-phototrophic compared to 7 g/L heterotrophic-phototrophic for *Haematococcus* spp. [61, 93, 101, 102]. Reduced production costs and time may drive *C. zofingiensis* into the high value market for astaxanthin production despite the quantity of astaxanthin being lower per cell [9, 10]. Further detailed information about astaxanthin in *C. zofingiensis* is reviewed by Liu et al. [103].

To become commercially competitive, astaxanthin extracts may need to be purified, adding cost to the process but this could be recuperated by the increased market value. Pure astaxanthin can be worth $15,000/kg for use as nutraceuticals, cosmetics, and food additives and the market size for this is $40 million [5]. For impure astaxanthin, the value can be up to $2000/kg which can be used as a feed additive for aquaculture, as a food colourant stabiliser, or as cosmetics [104]. Furthermore, the global astaxanthin market size was $359 million in 2019 [51] (Table 1).

### Lipids

In general, there is a lack of definition and distinction between the different types of algal lipids and the nomenclature often overlaps. Lipids can be distinguished into two overarching categories: membrane and intracellular (Fig. 2). Intracellular lipids are largely neutral and will be referred to as such throughout this section. The lipids that accumulate in *C. zofingiensis* have been researched for their potential use in biofuels and nutraceuticals [9, 105]. Total lipids can account for 65.8% DW of the total biomass [106] and this species produces a significant quantity of TAGs up to 40% DW [75, 105]. The cost of oil production from microalgae is 3–4 times higher than that of plant oils ($2.4/L in 2006) [107]. However, this price may decrease if algae are cultured mixotrophically or heterotrophically, $0.9/L, due to the higher biomass concentrations [108].

The type of lipid available depends on the culture conditions. In total, 70.6% of total lipids were membrane lipids (glycolipids and phospholipids) under phototrophic conditions, whereas 80.9% of total lipids were neutral intracellular lipids under heterotrophic conditions [109]. Glycolipids and phospholipids represent 12.95% and 1.92% DW in *C. zofingiensis* under nutrient-replete conditions [83]. It is generally inferred that there is more value in neutral lipids for biodiesel than membrane lipids, although use as functional food ingredients, pharmaceuticals such as anti-inflammatories, and surfactants is worth consideration [77–79]. However, if adding glucose to obtain astaxanthin, the quantity of membrane lipids decreases so this may not be economically viable [20]. Alternatively, there may be more value in the use of neutral lipids as nutraceuticals or food and feed additives rather than biofuels which are still not economically viable due to competition with petroleum oil, shale oil, and the large tracts of land required.

The fatty acid composition varies greatly depending upon the culture conditions and harvest time. Although challenging to define, total fatty acids (TFAs) generally consist of polyunsaturated fatty acids (PUFAs), monounsaturated fatty acids (MUFAs), saturated fatty acids (SFAs), and fatty acid methyl esters (FAMEs) [46]. Neutral lipids can account for up to 89.1% of the TFA pool [46]. ATCC 30,412 produces fatty acids mainly in the form of C16:0, C18:1, and C18:2 [110]. Table 2 demonstrates discrepancy between interpretation and analysis of data throughout research papers. EPA and DHA are not often found in the fatty acid profiles of *C. zofingiensis* [75, 111]. However, Cheng et al. [84] found 0.58% and 0.74% of the TFAs were EPA and DHA. There could be potential in investigating methods to enhance the content of EPA and DHA for extraction as these hold a high market value, although it may be preferable to consider more naturally abundant lipids for incorporation into the biorefinery process.

**Table 2:** Examples of the variation in fatty acid composition in *C. zofingiensis* cells under different culture conditions

Culture condition	Cultivation time	Lipids	Quantity	Reference
Glucose + N deprivation	8 days	TFAs Glycolipids	Increased Decreased	[20]
Phototrophic to heterotrophic	14 days	PUFAs in TAGs MUFAs SFAs	47.2 to 36% 25.1 to 41.2% 27.7 to 22.8%	[109]
N deprivation N deprivation + high light	6 days	PUFAs in TAGs MUFAs SFAs	25.7% TFAs 62.7% TFAs 34% TFAs	[24]
Heterotrophic	8 days	C16:0, C16:2, C18:1, C18:2, and C18:3	> 85% TFAs	[112]
Phototrophic Mixotrophic Heterotrophic	14 days	C16:0 C18:1 C18:1	28.33% TFAs 42.3% TFAs 37.33% TFAs	[70]
Any	10 days	C18:1 C18:2 C16:0	32.2–35.8% TFAs 18.2–20.1% TFAs 16.1–18.5% TFAs	[110]

### Carbohydrates

**Fig. 3:**
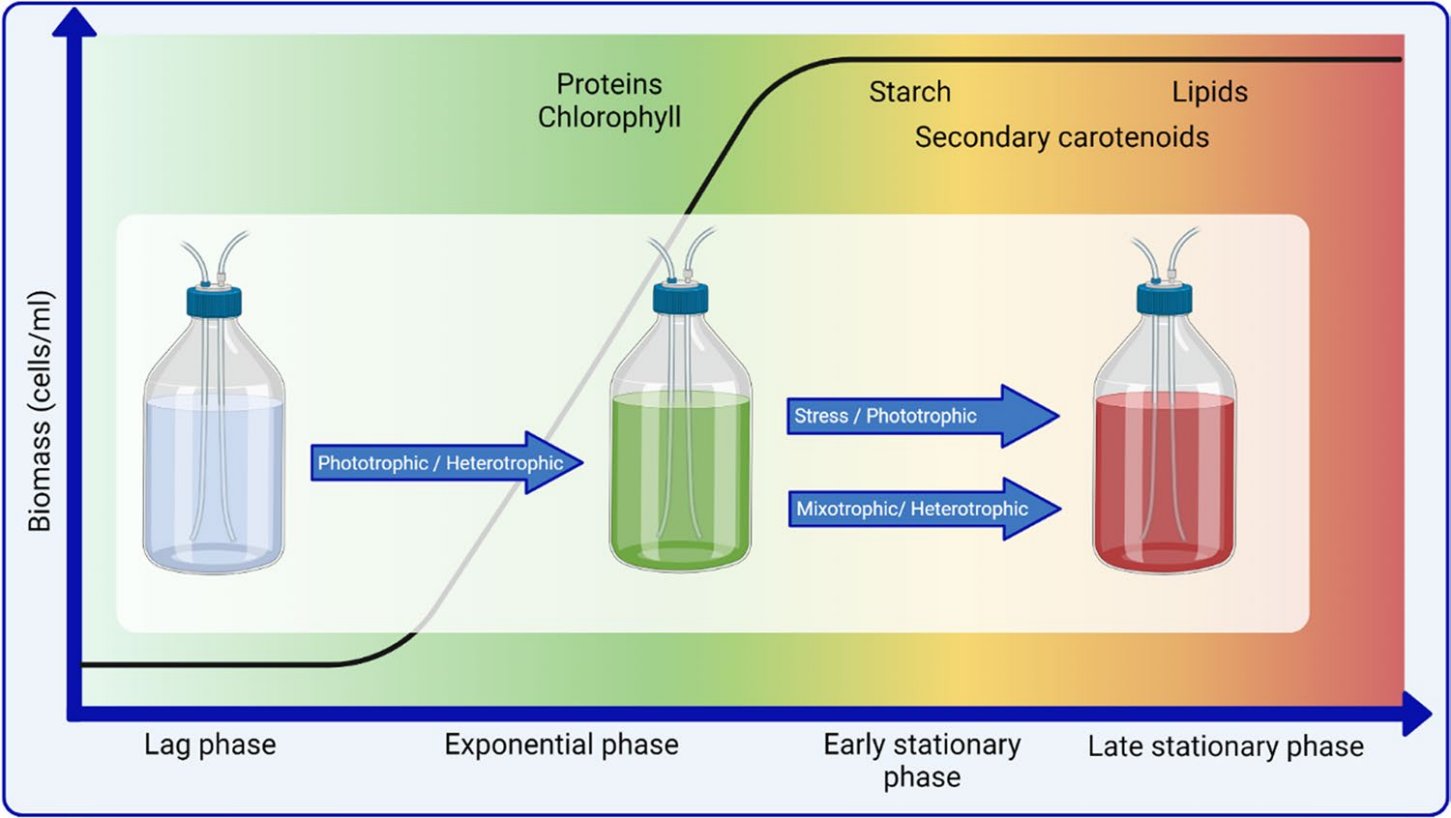
The *C. zofingiensis* general growth curve, indicating the change in culture colour (green to red) with time, growth mode, and growth phase. The timing of accumulation of various biochemical compounds within the culture is also shown

There is a balance between starch and lipid presence because they are the two dominant energy storage forms and share common carbon precursors for biosynthesis [110]. There is limited research on the polysaccharides produced by *C. zofingiensis*, but total carbohydrates, starch, and EPS have been investigated. The highest reported carbohydrate contents in *C. zofingiensis* have been 47.7% and 66.9% DW, both obtained under nitrogen deprivation [25, 83]. Starch, which could be easily separated in a biorefinery procedure due to its high density and insolubility, accumulated in mixotrophic glucose-fed ATCC 30,412 cultures in greater concentrations than in heterotrophic cultures [110]. Starch accumulates before lipids under such conditions because lipids require more energy than starch for production (Fig. 3). Starch can represent 66.7% of the total carbohydrates and has good potential as a bioethanol feedstock or for bioplastics [25, 26]. Stationary phase cells from phototrophically grown *C. zofingiensis* were studied as a source of fermentable sugars for second-generation bioethanol production. Following enzymatic hydrolysis, it was found that 58.1% of the total reducing sugars and 95.3% of the total hydrolysed sugars were glucose which could have been derived from cell wall cellulose or starch granules [113]. Mixotrophically grown ATCC 30,412 has also produced EPS at 208.4 mg/L which had inhibitory effects on cancer cell viability and exhibited radical scavenging activities and consisted of ten different monosaccharides or their derivatives [39]. Although the more EPS that was produced, the less viable the cells were, which could be problematic when aiming to valorise the whole biomass [39]. Furthermore, EPS may cause difficulties in obtaining and separating other components and could be problematic in terms of fouling of equipment during cultivation and harvesting. Nonetheless, the value of EPS may exceed that of starch and have more beneficial applications such as pharmaceuticals. Other types of carbohydrates include cellulose, glucose, and xylose that form the structure of the cell walls. So, if mild cell disruption is possible and cell walls can be separated then such polysaccharides could be recovered. Overall, there is potential for EPS or starch to be one of the extracted components from *C. zofingiensis* as part of a biorefinery approach although there may be more value in obtaining higher concentrations of lipids rather than carbohydrates.

### Proteins

Proteins from microalgae have many applications most commonly as food and feed additives (Table 1). There is little information available about the proteins and amino acids of *C. zofingiensis* likely due to the focus on increasing TAGs and astaxanthin, which causes a reduction in protein content [1]. The highest protein content recorded for SAG 211–14 biomass is 48.4% where the nitrogen source was ammonium nitrate [114]. Protein content also varied under different light intensities, 13% at 300 µmol/m^2^/s and 20% at 50 µmol/m^2^/s which could imply that excess light induces protein degradation or that nitrogen uptake is better at lower light intensities [115]. Even if the protein content is a minor fraction of the biomass, ~ 10% of a product at large scale can provide a substantial amount of revenue. The only amino acid data for *C. zofingiensis* is for ATCC 30,412 and is available in the supplementary data from Liu et al. [116], which shows the variation in amino acids under nitrogen deprivation. All essential amino acids were present except for tryptophan although shikimic acid was reported which is involved in tryptophan biosynthesis. Tryptophan is often undetected from certain methods of amino acid analysis. Therefore, studies employing methods which can fully qualify all amino acids should be conducted with this species and the concentrations compared to other known protein sources which will affect whether it can be used as a feed supplement independently.

Novel proteins such as caleosins, lipases, and dehydrin-like proteins have been identified in *C. zofingiensis* which contribute to homeostasis and prevent protein denaturation caused by ROS accumulation [117, 118]. Proteins with molecular masses ranging from 38 to 1932 kDa have been identified which should be considered when separating proteins from other cellular components, for example, via membrane filtration [118, 119]. As well as using the proteins directly, there is scope for extruding meat analogues from heterotrophically grown microalgae which could be worth investigating in *C. zofingiensis* for sustainable meat alternatives [120]*.* Further research could also investigate the production of enzymes for pharmaceutical applications which hold high value and require low volumes [121].

### Additional components with potential value

There are other more specific molecules that may carry commercial value. *C. zofingiensis* possesses the enzymes necessary for biosynthesis of abscisic acid, a plant growth hormone, which could have use as a biostimulant for plants [122]. SAG 221–2 was described as having an alpha-tocopherol (vitamin E) content of 182.2 µg/g DW in the stationary growth phase and 343.32 µg/g in the stationary phase under nitrogen limitation [123]. The total phenol contents for phototrophic and mixotrophic ATCC 30,412 cultures were 40.8 µgGAE/mg and 15.1 µgGAE/mg (gallic acid equivalents) which had antioxidant activities of 13% and 14% and the ferric reducing antioxidant power’s were 9.29 µM AAE/mg (ascorbic acid equivalents) and 1 µM AAE/mg respectively [33].

## Effect of culture conditions, stress, and trophic mode on cellular composition

Most of the research on this species investigates the effect of culture conditions and growth modes on cellular composition. Given a large number of variables on different strains of interest, a wealth of literature is available providing a broad range of results. To distil this information and frame it in the context of a biorefinery approach, the data has been categorised into the different growth modes each of which can be used to obtain high quantities of carotenoids and lipids, which are both desirable as part of a biorefinery process for this species (Fig. 3). Stressed phototrophic and mixotrophic or heterotrophic cultivation are unfavourable for algal growth, although the biomass still increases due to the accumulation of storage compounds rather than cell division [75, 83, 124]. The benefit is that desirable components, such as astaxanthin, accumulate in the algal cells. To obtain the red phase in *C. zofingiensis*, a two-stage approach is required, except for when the stationary phase of stressed phototrophic cultures is used. Two stages may require additional equipment, cleaning, and energy so cultivation duration versus quantity of product should be considered. When deciding upon which method to use to optimise the biomass productivity, composition, operating expenses (OPEX), and the risk of contamination should be considered. The strengths and weaknesses of each growth mode are compared in Table 3.

**Table 3:** Growing *C. zofingiensis* in different growth modes for accumulation of carotenoids and lipids [9, 62, 71, 101, 109, 125–127]

Growth mode	Cost of carbon source	Contamination risk	Two-stage costs	CAPEX/OPEX of lighting	Biomass accumulation	Astaxanthin accumulation (per cell)	Lipid accumulation (per cell)	Time efficiency
Phototrophic	None	Low	None	High	Low	Low	Highest	Slow
Mixotrophic	High	High	Yes	High	High	Higher	High	Fast
Heterotrophic	High	High	Yes	None	High	High	High	Fast
Heterotrophic-phototrophic	High	High	Yes	High	High	Highest	n/a	Fast

### Biomass production strategies

The biomass concentration varies hugely depending on the culture conditions, starting biomass concentration, and the cultivation strategy. The highest cell densities recorded for *C. zofingiensis* are 13.5 g/L phototrophically [125], 10.71 g/L mixotrophically [70], 98.4 g/L heterotrophically [101], 221 g/L fed-batch heterotrophically [126], and 73.7 g/L using a heterotrophic-phototrophic two-stage approach [61]. Concentrating the biomass prior to applying the stressed conditions may be beneficial in terms of the volume and reduction of bacterial contamination [127], but the necessary light intensity per cell should be known so that self-shading does not prevent carotenogenesis. Based on the research so far the most promising technique for biomass and astaxanthin production from *C. zofingiensis* was using a heterotrophic-phototrophic approach [61].

The type of system used for algal production is important as it will affect the efficiency and economics of a biorefinery process. The most cost-effective cultivation method is to employ raceway ponds, where the price for by-products can be $5/kg which covers the total cost of raceway production, meaning that the revenue obtained from the target product may be considered as profit [104]. Raceways, however, are open to the environment, offering little control over (a)biotic conditions, water loss, or axenicity. Photobioreactors (PBRs), on the other hand, are more reliable and allow control over abiotic factors, the precision of which will depend on whether the system is inside or outside and the geographical location [128]. The scale-up of such systems should be further researched as it has been evidenced that differences occur when scaling from lab to PBR. Some drawbacks with PBRs can be light availability as the culture grows, temperature control, and the intensive capital required for construction and maintenance [9]. The high cell density accumulation of *C. zofingiensis* could mean that achieving high light per cell for product accumulation may be impaired so the type of system must also be optimised. The light source needs to be reliable so outdoor cultivation may not be suitable in certain geographies and seasons despite sunlight being a free source of energy. LEDs are expensive to invest in and although the energy usage is lower than traditional lighting, costs may still be high or not provide adequate light quality. Even in Iceland where energy is fully renewable and available at low expense, lighting is the most significant cost of the process [129]. Additionally, if using heterotrophy then the fermenter system will also need to be optimised for biomass and product accumulation [9, 61].

### Abiotic factors

Microalgal growth and biochemical composition are strongly influenced by biotic and abiotic factors which are often interrelated. The most successful conditions for biomass accumulation and induction of carotenoid and lipid accumulation in *C. zofingiensis* are summarised in Table 4, although it is worth noting that conflicting findings in the literature are a result of varying combinations of abiotic factors. The growth medium has a significant impact on the cellular composition of microalgae. Several authors have explored the effect of the nitrogen source on *C. zofingiensis* and have concluded that replacing nitrates with urea is a realistic, cheaper alternative because although it causes a 32% lag in productivity, nitrate can be eight times more expensive and can account for 80% of the total growth medium cost [130–133]. Higher concentrations of urea caused increased biomass concentrations (8.86 g/L with 3.81 mmol/L urea) but a lower accumulation of carotenoids [134]. However, when the urea concentration was lowered (0.19 mmol/L), the highest carotenoid content and yield (0.99 mg/g, 4.11 mg/L) were reached due to a high C:N and the astaxanthin content was not substantially affected or inhibited [134]. Urea also increased lipids in CALU-190 by 30.6% compared to nitrate and did not alter the carotenoid (0.3% DW) composition or fatty acid profile which was compatible with the current European biodiesel standards [62].

**Table 4:** Successful conditions for biomass accumulation and stress of *C. zofingiensis*

Abiotic factor	*C. zofingiensis* biomass accumulation	Induction of red phase in *C. zofingiensis*	Reference
Temperature	20–30 °C	24 °C astaxanthin 25–30 °C astaxanthin 28 °C lutein	[9, 67, 93]
pH	5.5–8.5	5.5 astaxanthin 6.8–7.2 lipids and FAMEs High or low extremes	[9, 15, 135]
CO_2_	1–5%	5%	[9, 10]
Light intensity	250–1000 µmol/m^2^/s or 28–45µE g-ds/s per cell	High 350 µmol/m^2^/s 300 µmol/m^2^/s	[92, 101, 136]

### Phototrophic stress conditions

*C. zofingiensis* accumulates lipids and carotenoids simultaneously under stress-inducing conditions such as high light, low nutrients, or high salinity. Bar et al. [37] suggest that the response of UTEX 32 to stress occurs in three stages: (1) primary and secondary carotenoids protect the photosynthetic apparatus against photooxidation, (2) primary carotenoids reduce and secondary carotenoids accumulate to protect photosynthesis, (3) a lipid layer containing secondary carotenoids surrounds the cell to form a hydrophobic light filter to reduce irradiation, prevent formation of oxygen radicals, and reduce water loss.

Increasing the light intensity and depriving *C. zofingiensis* cultures of nutrients have increased production of carotenoids, lipids, and starch [2, 49, 92, 137]. Nutrient deprivation may be achieved when a growing culture reaches the stationary phase (Fig. 3) or via concentration and washing of the cells then replacement with nutrient-deprived media. High light intensity incurs additional costs and greater energy inputs which will only increase with the density of the culture.

Secondary carotenoids have been detected as soon as 1 h after light stress (300 µmol/m^2^/s) and nitrogen depletion [37]. This could be because two genes encoding β-ketolase, the key enzyme synthesising astaxanthin, are upregulated by high light [138]. Nitrogen deprivation plus high light gave UTEX 32 the highest astaxanthin and TAG productivities, 3.3 mg/L/day and 297 mg/L/day, compared to cultures under low light, high light, and nitrogen deprivation individually [24]. When comparing nitrogen, phosphate, and sulphur deprivation on ATCC 30,412, the highest astaxanthin (0.624 mg/L/day) and TAG (27% DW) contents were achieved under nitrogen deprivation [139]. Additionally, the effect of 0.04%, 1.5%, and 5% CO_2_ on nitrogen-deprived cultures caused increasing concentrations of astaxanthin (1–3 mg/g) during 96 h of culture [10]. Furthermore, the combination of nitrogen and phosphorus deprivation increases total lipid production and FAME yield compared to individual deprivation of such elements [140]. When grown outside in natural sunlight, the lipid content of nutrient-deplete *C. zofingiensis* was 54.5% DW compared to 27.3% DW in nitrogen-replete controls [141]. Under nitrogen deprivation, the lipid composition also changes; neutral lipids increased to 86.7% of the total lipids and TAG content was 27.3% DW, which was three times higher than the control [83]. After 1 day of nitrogen starvation, total carbohydrate and starch increased by 37% and 4.7-fold, respectively [25]. Such examples further demonstrate that the composition of the algal biomass is influenced by the culture conditions which can be optimised to provide the most desirable components for biorefinery.

### Mixotrophic culture

The effect of mixotrophy was first observed in *C. zofingiensis* when cultures turned from green to orange or red in the presence of glucose or sodium acetate in the light [142]. Photosynthesis and glucose metabolism operate in a dynamic balance during mixotrophic cultivation: the enhancement of one leads to the lowering of the other [143]. Regulating C:N by adding a carbon source under excess light improves the content of astaxanthin and TAGs whilst the biomass concentration still increases due to cell volume rather than the number of cells [45, 124]. Glucose represses photosynthetic pathways and upregulates ketocarotenoid biosynthesis and heterotrophic carbon metabolism [45, 144]. Therefore, under mixotrophic conditions chlorophyll *a* and chlorophyll *b* concentrations decrease and carotenoid concentrations increase where astaxanthin can account for 60% of the total pigments [14, 33, 145]. Mixotrophic cultivation with glucose has been considered to be better than heterotrophic culture in terms of biomass (2.36 g/L/day, 63% higher than heterotrophic) and starch accumulation but inferior in terms of fatty acid concentrations [146].

Mixotrophic cultivation with low nitrogen has been evidenced to increase astaxanthin productivity by 3.4 times at 300 µmol/m^2^/s and 3.9 times at 50 µmol/m^2^/s compared to heterotrophy [124]. When comparing glucose concentration and nitrate concentration, 30 g/L and 0.55 g/L were optimum for astaxanthin yield respectively [147]. In a comparison between mixotrophy and heterotrophy with and without nitrogen, mixotrophy without nitrogen had the highest astaxanthin and total carotenoid content, 0.24% DW and 0.4% DW respectively [101]. Contrastingly, culturing ATCC 30,412 in a PBR with high light, nitrogen deprivation, and glucose did not improve the astaxanthin, canthaxanthin, or adonixanthin concentration compared to cultures without glucose. But, the TFAs and neutral lipids were higher for glucose cultures and phospholipids and glycolipids declined [20]. Still, astaxanthin did increase over time just at a lower rate with glucose so the balance between astaxanthin, neutral lipids, and biomass accumulation should be further investigated. Furthermore, the highest total lipid content recorded in mixotrophic conditions (nitrogen starvation, 300 µmol/m^2^/s, 5 g/L glucose) was 42.4% [115].

### Heterotrophic cultivation

*C. zofingiensis* can grow heterotrophically showing potential for fermentation production [19, 148]. The effect of heterotrophy was first observed when cultures turned from green to orange or yellow in the presence of glucose, potassium acetate, or sodium acetate in the dark [142]. The benefit of heterotrophy over mixotrophy is that the energy cost associated with the provision of lighting is negated [149]. However, although *C. zofingiensis* can achieve high cell density heterotrophically, the intracellular astaxanthin content can be relatively low compared to phototrophic stress or mixotrophy [103]. Nonetheless, if the correct conditions are employed, the high biomass productivity may be able to counteract this.

Heterotrophic cultures of *C. zofingiensis* can achieve a comparable astaxanthin yield to *H. pluvialis* on a volumetric basis [103]. There are many examples of the heterotrophic growth mode with glucose increasing astaxanthin in *C. zofingiensis* [100, 149, 150]. High glucose and low nitrate are optimal for astaxanthin production in batch cultures [89, 91], although a higher astaxanthin yield of 11.14 mg/L was obtained from fed-batch culture with a combined glucose–nitrate mixture [91]. Moreover, a two-stage heterotrophic strategy where the media changed from 46.7, 1.13, and 0.125 g/L to 35.2, 0.281, and 0.023 g/L glucose, nitrate, and MgSO_4_·7H_2_O, respectively, revealed that the highest astaxanthin yield (0.96 mg/g or 15.1 mg/L) was 74% higher than that in standard media [29]. After fermentation with 20 g/L glucose, the highest astaxanthin content reported was 73.3 mg/L or 0.07% DW at a cell density of 98.4 g/L which was considered to be comparable and even higher than quantities achieved from *H. pluvialis* [101].

Heterotrophy allows maximum accumulation of lipids but lower levels of astaxanthin and lutein compared to mixotrophy and phototrophy [70, 124]. The higher the glucose concentration the higher the lipid and TFA content in *C. zofingiensis* [74, 151]. Araya et al. [152] grew UTEX 32 phototrophically and found lipid productivity of only 10.95 mg/L/day, whereas Liu et al. [74] obtained 660 mg/L/day and 710 mg/L/day with glucose and molasses respectively. Heterotrophy with 30 g/L glucose caused an increase in lipid yield, from 25.8% DW to 51.1% DW, and accumulated predominantly neutral lipids (79.5%), 88.7% of which were TAGs [109]. Neutral lipids have also been found to account for 85.5% of the total lipids [74]. The benefits of lipid accumulation by heterotrophic growth are further evidenced by Liu et al. [112]. Fatty acids, especially C18:1, promoted the accumulation of astaxanthin esters and the fatty acid composition of astaxanthin esters was correlated with the TFAs [151]. C18:1 was promoted by higher sugar concentrations, whereas C18:3 was promoted by lower sugar concentrations [74, 112]. When fed with 30 g/L glucose, ATCC 30,412 synthesised lipids up to 0.531 g/g in the dark, compared to 0.352 g/g in the presence of light, but no significant difference was observed in the fatty acid composition which is distinct from the findings of other authors [110].

### Heterotrophic-phototrophic

Several authors have adopted two-stage strategies to further optimise the composition of *C. zofingiensis* biomass. A heterotrophic-phototrophic strategy achieved 194.5 mg/L or 2.7 mg/g astaxanthin which is the highest reported volumetric value for this species [61]. Another example, that used glucose in the first stage and high light in the second stage, enhanced the intracellular accumulation of astaxanthin to 3.5 mg/g, 3.2 times higher than under heterotrophic conditions, although this data is yet to be published [103]. A further study found the highest astaxanthin productivity and content, 5.26 mg/L/day and 0.11% DW, respectively, were achieved when the starting culture was diluted fivefold [101]. The high astaxanthin concentrations are promising; however, the increased time, costs, water usage, space, and downstream processing associated with a two-stage process must be carefully considered prior to scale-up.

### Addition of chemicals

Another method of stressing the cells is to add chemicals to the culture which has been demonstrated across all growth modes. For phototrophic conditions, the most frequently investigated is an increase in salt concentration. This was first evidenced by Borowitzka et al. [153] in both *C. zofingiensis* and *H. pluvialis* and has since been demonstrated to increase primary and secondary carotenoids or TAG concentrations in *C. zofingiensis* cells [16, 154–156]. In ATCC 30,412 cells, high light (150 µmol/m^2^/s) and NaCl stress caused increased ROS levels which upregulated carotenogenic genes enhancing biosynthesis of zeaxanthin, canthaxanthin, and astaxanthin [11]. The genes in starch degradation pathways were also upregulated under salt stress providing carbon building blocks via glycolysis for storage lipids [156]. Other chemicals that have been used to enhance the accumulation of carotenoids in phototrophic *C. zofingiensis* are pyruvate, citrate, malic acid, hydrogen peroxide, and sodium hypochlorite [157]. The addition of Fe^2+^ under mixotrophic conditions caused decreased growth by up to 32% but increased astaxanthin by 100% to 2.17 mg/g or 25.8 mg/L. Fatty acids increased by 41.8% to 5.87 g/L which was superior to *H. pluvialis* which only gave 0.2 g/L fatty acids due to the low biomass concentration [158]. The addition of phytohormones, indole propionic acid and indoleactetic acid, to mixotrophic ATCC 30,412 cultures has also successfully increased astaxanthin (89.9 mg/L or 7.5 mg/L/day) and lipid (65.5% DW or 445.7 mg/L/day) contents [159]. The addition of chemicals such as peroxynitrite and H_2_O_2_ to heterotrophic cultures has also demonstrated increases in carotenoids [160, 161]. Nevertheless, when minimising costs and prioritising the stability and quality of products such as astaxanthin, the effect of how chemicals are metabolised should be investigated. Furthermore, an additional stage may be necessary to wash algal cells prior to disruption as part of a biorefinery system because if chemicals exist in an unmetabolised form, interaction with released intracellular products could occur. This will result in an increase of freshwater use.

### Carbon source

If growing *C. zofingiensis* mixotrophically or heterotrophically, the carbon source is important. Generally, the higher the sugar concentration, the higher the cell density but the lower the specific growth rate is due to substrate inhibition [74, 162]. Glucose is the most commonly used carbon source for mixotrophic and heterotrophic cultures of microalgae because higher rates of growth and respiration are obtained [162]. The effect of glucose on *C. zofingiensis* carotenogenesis should be attributed to the consequence of sugar metabolism rather than to osmotic stress [91]. Alternative and waste carbon sources have been investigated to make the process economically viable whilst improving sustainability and moving towards a circular economy [103, 109]. For example, under mixotrophy and heterotrophy, molasses has been shown to improve biomass, astaxanthin, and lipid accumulation compared to glucose [74]. However, the use of waste streams may also not be beneficial when aiming to obtain high-purity compounds such as astaxanthin that can compete in the current market. Therefore, the use of waste may not be necessary if establishing a cost-effective biorefinery approach where multiple valuable components are separated.

Overall, there is a wealth of research with arguments for and against each cultivation mode. When creating a biorefinery process, the input costs must be assessed and established based on the potential profit. To obtain the highest quality products, indoor PBRs or fermenters will allow the most reliable products although the use of energy and recycling within the system will need to be carefully tailored. The use of two stages is necessary for almost all instances, but the red phase can be initiated using the phototrophic stationary phase. Mixotrophy and heterotrophy cause chlorophyll *a* and *b* to decrease which could allow easier separation of products in the downstream process due to reduced interference. Phototrophic cultivation has been considered the best for lipid accumulation, whereas the heterotrophic-phototrophic two-stage method gave the highest biomass and astaxanthin concentrations. When considering the type of sugar, the efficiency of the sugar source is essential as the longer the transition from green to red, the higher the risk of contamination. When deciding on the conditions to employ to acquire biomass with a desirable composition for biorefinery, the advantages and disadvantages must be carefully considered based on how the products are being prioritised (Table 3).

## Biorefinery

Some areas of biorefinery have been investigated for acquiring products from *C. zofingiensis*, but there is a need for optimisation of each stage as well as testing each of these in succession. Each method depends upon the target products, previous treatments, the required purity and form, the scale, and the biochemical composition of the algae. The harvesting method should be the most efficient in terms of energy, time, and percentage of biomass obtained and this stage influences the later stages of the downstream process. Breaking the cells correctly is imperative for obtaining the products so investigation into the cell wall structure at different growth stages and cell disruption techniques that are energy efficient is necessary. The fractionation step that follows must be considered carefully to prevent the mixing of products which could interact and become impossible to separate. The order in which products are obtained must be considered throughout the process to ensure that the higher value components such as astaxanthin are not compromised. Ideally, the downstream processing facilities will be in the same location as the upstream process facilities to ensure efficient processing and to reduce storage and transportation costs and emissions.

### Harvesting

The aim of harvesting is to separate the maximum number of cells from the liquid media. Harvesting microalgae can be difficult and costly (14–30% of production costs) due to the small cell size [163, 164]. Harvesting is the most widely investigated aspect of downstream processing for *C. zofingiensis* specifically centrifugation, flocculation, coagulation, and flotation. Improving this step could greatly advance the feasibility of a biorefinery. The harvesting step must concentrate the algal biomass whilst limiting damage and without altering the composition and this must be achieved at a low cost. The harvesting method used can affect the biochemical and elemental profiles of the algal biomass. For example, the protein content was 47.4%, 30.5%, and 48.4% DW while the carotenoid content was 0.79%, 0.37%, and 0.76% DW when centrifugation, sedimented non-neutralised centrifugation, and sedimented neutralised (to pH 7) centrifugation were compared on SAG 211–14 [114]. This could be because certain salts and hydroxides precipitated with the biomass when the non-neutralised technique was used causing an increase in ash content.

Research into harvesting methods of *C. zofingiensis* has demonstrated that the most effective mechanisms are flocculation and coagulation. The most significant research on flocculation was by Mayers et al. [114] and Wyatt et al. [165] where both an NaOH and Mg^2+^ combination and FeCl_3_ achieved high flocculation efficiencies of 89.4% and 90%. It is suggested that bio-flocculants should be studied to determine the influence on the quality of other products despite their efficiency being reduced compared to regular flocculants [166]. For example, PEI-coated *Escherichia coli* that had been exposed to UV irradiation was used to harvest *C. zofingiensis* and gave 53% recovery efficiency, 70% harvesting efficiency, and 38% recovery capacity [167]. Moreover, Guo et al. [168] produced low-cost bio-flocculants from untreated corn stover using biomass-degrading bacteria *Pseudomonas* sp. where the highest flocculant efficiency for *C. zofingiensis* was 77.9%. For coagulation, Zhang et al. [169] used Al^3+^ with dissolved air flotation and obtained a maximum harvesting efficiency of 90% regardless of the *C. zofingiensis* growth phase. Furthermore, harvesting efficiency increased with coagulant dosage under different conditions with the most efficient being 91% with 250 mg/g Fe^3+^ at pH 8 [170]. Later, Mg^2+^ coagulation was shown to be more effective than Fe^3+^, Al^3+^, or chitosan for dissolved air flotation where the maximum harvesting efficiency was 94% [171]. However, it is important to recognise that coagulation will affect the ease of cell disruption because the cells are clumped together making it more difficult to target individual cells. Alternatively, Gerulova et al. [172] aimed to save energy by using magnetite nanocomposites where the highest harvesting efficiency for SAG 211–14 was 95% at pH 4 with 200 mg/L Fe_3_O_4_–PEI. To select the most robust and sustainable option the pH, initial culture density, toxicity of the flocculant, flocculant/coagulant dose, final use of the biomass, and the costs should be considered. Coagulation is already applied at a large scale for wastewater treatment so it may be a viable option depending on the target products.

It has been advised that centrifugation is preferable to flocculants or coagulants, despite the higher recovery rates, to obtain pure products because such harvesting methods lower the value of the products [166, 173]. When filtration and centrifugation are used, the quality of the biomass must be high because these have prohibitively high operating and capital costs preferable to the production of high-value products such as astaxanthin [173]. However, technologies such as membrane filtration can be used for multiple purposes in up- and downstream processing so the investment can be a beneficial and a cost-effective way of maintaining high quality. Research into such methods for *C. zofingiensis* should therefore take place.

### Cell disruption

**Fig. 4:**
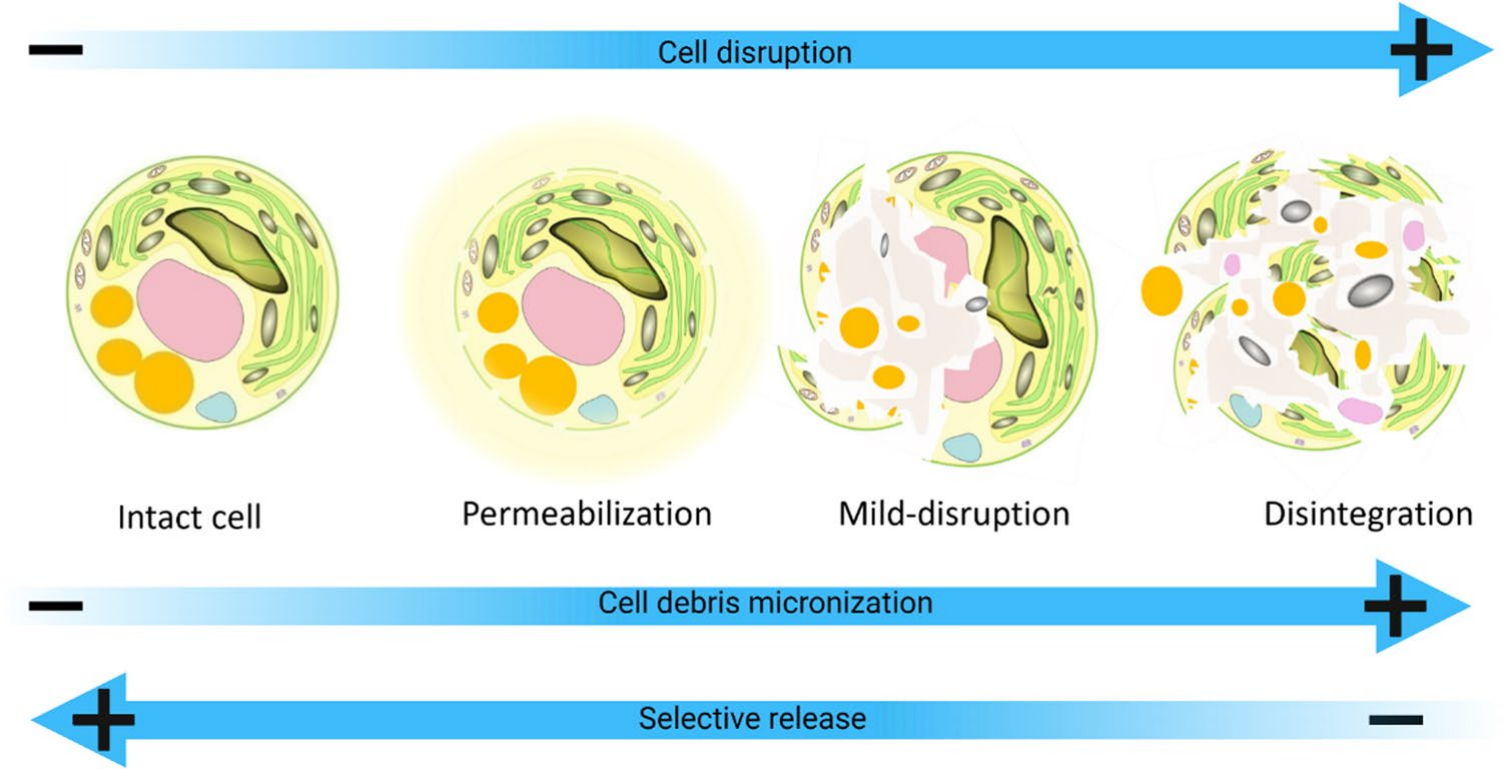
The interaction between the level of cell disruption and release of cellular components from microalgae in general [183]

After concentration, the next stage in a biorefinery process is cell disruption to selectively release the components within the cells (Fig. 4). There are two main options here, the dry route or the wet route. The dry route for biorefinery is a more traditional method whereby the biomass is first dried, which can disrupt or partially disrupt the cells, and then desirable compounds are extracted using solvents based on hydrophobic/hydrophilic properties [174]. The effect of cell disruption from drying may be limited if the optimised biomass has a thick cell wall. There are different methods including freeze, spray, drum, solar, cross-flow air, vacuum shelf, flash, convective, fluidised bed, and incinerator drying with the first four being the most common [175–177]. Such methods can be beneficial if the biomass needs to be stored or transported. It is necessary to optimise drying conditions to preserve the biomass in its most functional form and the type of drying technology selected depends on the desired end products [176, 178]. Nonetheless, processes for refining wet biomass, which can be concentrated into a paste or slurry, may have significant advantages, as energy costs can be saved [5].

The wet route uses techniques to selectively disrupt the cell wall directly after harvesting. This idea was introduced by Halim et al. [179] and Jubeau et al. [180]. Halim et al. [179] describe the process used by OriginOil Inc. for extracting oil from microalgae for biofuels. Jubeau et al. [180] selectively disrupted *Porphyridium cruentum* to obtain proteins and phycoerythrin. The water acts as the solvent during cell disruption; and therefore, this stage also incorporates extraction of certain soluble target compounds. The milder the cell disruption process is, the higher the selectivity of products. As cell disruption becomes harsher, the finer the particles of cell debris become (micronization) (Fig. 4). If disruption is too harsh, the components can be damaged or can interact together making separation difficult. Therefore, optimisation should take place to find the perfect balance between the level of disruption and product recovery [181]. The cell wall and cell structure should also be considered when applying disruption techniques [182]. For *C. zofingiensis*, the thick cell wall during the red phase may require harsher treatments, but this is still possible without total disintegration of all parts of the cell. Certain techniques can be applied in both mild and harsh forms allowing exploration of the level of disruption required.

Research for cell disruption of *C. zofingiensis* has focussed on total disruption of the cell or disruption to obtain or analyse one specific product rather than to obtain multiple products. The culture conditions, the physiological state of the cell, and harvesting/dewatering methods will all influence the cell disruption efficiency. As with *Haematococcus* sp., the red cell stage of *C. zofingiensis*, which is desirable, causes increased cell wall strength making it more difficult to break. Taucher et al. [30] found the highest disruption yields for freeze-dried SAG 211–14 when ball milling (2.81 µg/g total carotenoid DW) and high-pressure homogenization (2.87 µg/g total carotenoid DW) were used, compared to ultra turrax (1.68 µg/g total carotenoid DW). But, when freeze-thawing, sonication, and freeze-drying were tested, the carotenoid yield was not determined and therefore such methods should be investigated further or in conjunction with other methods. Additionally, Araya et al. [152] found that ball milling freeze-dried UTEX 32 was 1.7 times more effective for the recovery of lutein than glass bead vortexing. It is important that future research investigates the methods in terms of mild cell disruption: breaking the cell wall but avoiding damaging organelles or causing mixing of products that will be difficult or impossible to separate. Future research must also focus on improving the understanding of the relationship between cell wall disruption mechanisms and cell wall composition and structure at different growth stages, as well as optimising the energy consumption to improve product recovery enough to balance the economics [43]. Furthermore, investigation and optimisation of cell disruption methodologies at different scales could be key for unlocking the potential of *C. zofingiensis* biorefineries. Separation following cell disruption must be investigated to acquire knowledge on product interaction and debris composition.

### Fractionation

Novel or improved separation and extraction systems with higher efficiencies can increase product recovery and provide promising results for future applications [162]. Research into a specific separation of multiple products from one source of biomass has not been explored for this species despite it being repeatedly referred to as a suitable candidate for biorefinery and this, therefore, requires detailed investigation. It has been studied for single products from a research perspective but not at a commercial scale. In general, processing and purification costs of microalgal products are poorly assessed at scale and will vary from strain to strain and scenario to scenario. Biorefinery approaches are essential for improving the economic balance of the production processes and need to be developed and tested for various product categories and strains [5]. In theory, after a disruption step, you will have a complex mixture with insoluble particles, i.e. debris, soluble molecules, and insoluble molecules. It may be necessary to include sequential fractionation steps to obtain fractions and extracts suitable for further purification. The only example of fractionation of *C. zofingiensis* is an efficient high-speed counter-current chromatography (HPCC) method for the separation and purification of canthaxanthin [63]. Canthaxanthin at 98.7% purity from 150 mg crude extract (2.1% canthaxanthin) was obtained by solvent extraction, in a one-step separation, and its recovery was 92.3% [63]. Nonetheless, one can learn from techniques used on other species, where similar products have been targeted. For example, the use of solvents and membrane filtration processes have been shown to recover 94% of polar lipids, 85% of carotenoids, and 86% of glycerol from *Dunaliella salina* biomass [184]. For *C. zofingiensis*, the separation of astaxanthin from TAGs may be difficult and costly because carotenoids are lipophilic molecules. However, the high potential profit may make this economically viable. Alternatively, ingestion of lipids can increase absorption of astaxanthin implying that separation from astaxanthin from TAGs may not be essential and that focus can be placed on other by-products from the cell debris and lipophobic phases [53]. Nevertheless, separation of the two component types is desirable to increase the value of the end products and to tap into a broader range of applications. Analysis of cell debris components is also necessary to identify the other by-products that can be obtained post cell disruption.

### Conceptual design

**Fig. 5:**
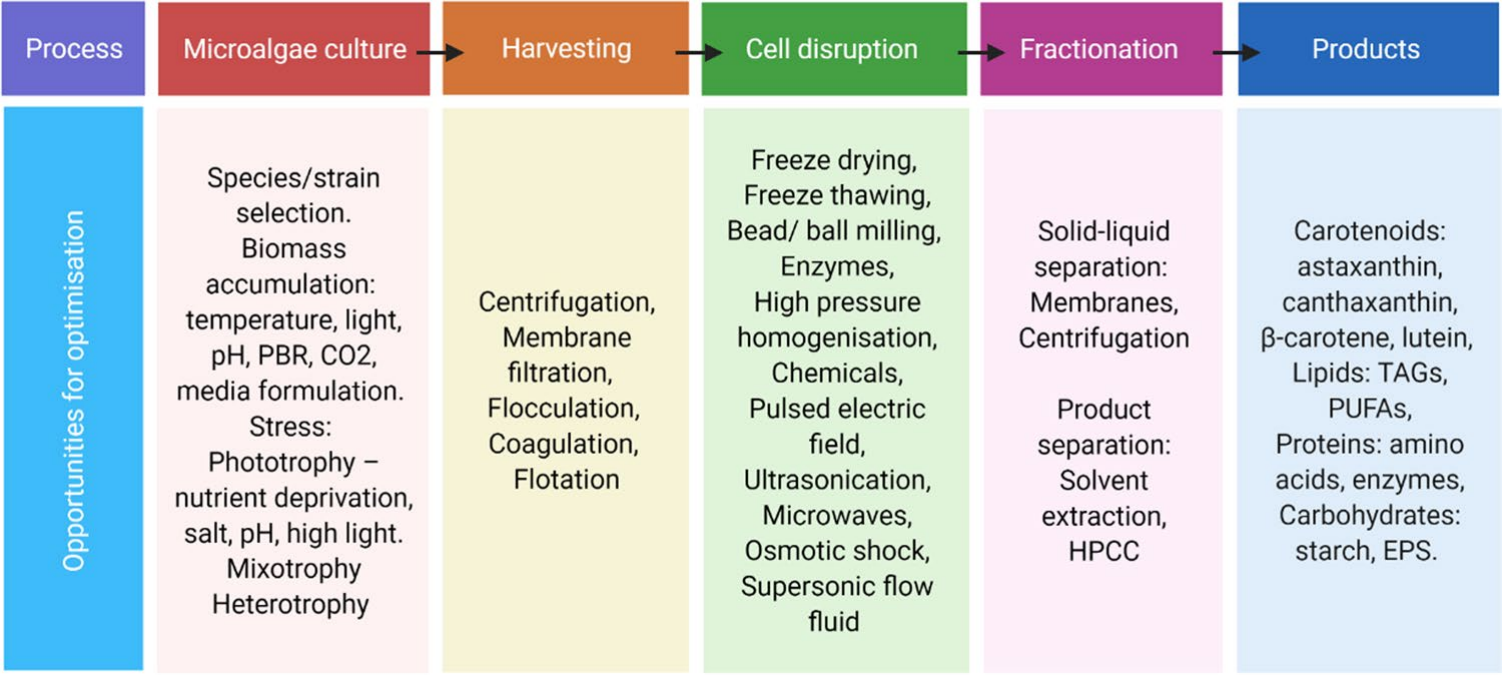
Opportunities for optimisation at each stage of the biorefinery process for *C. zofingiensis*

**Fig. 6:**
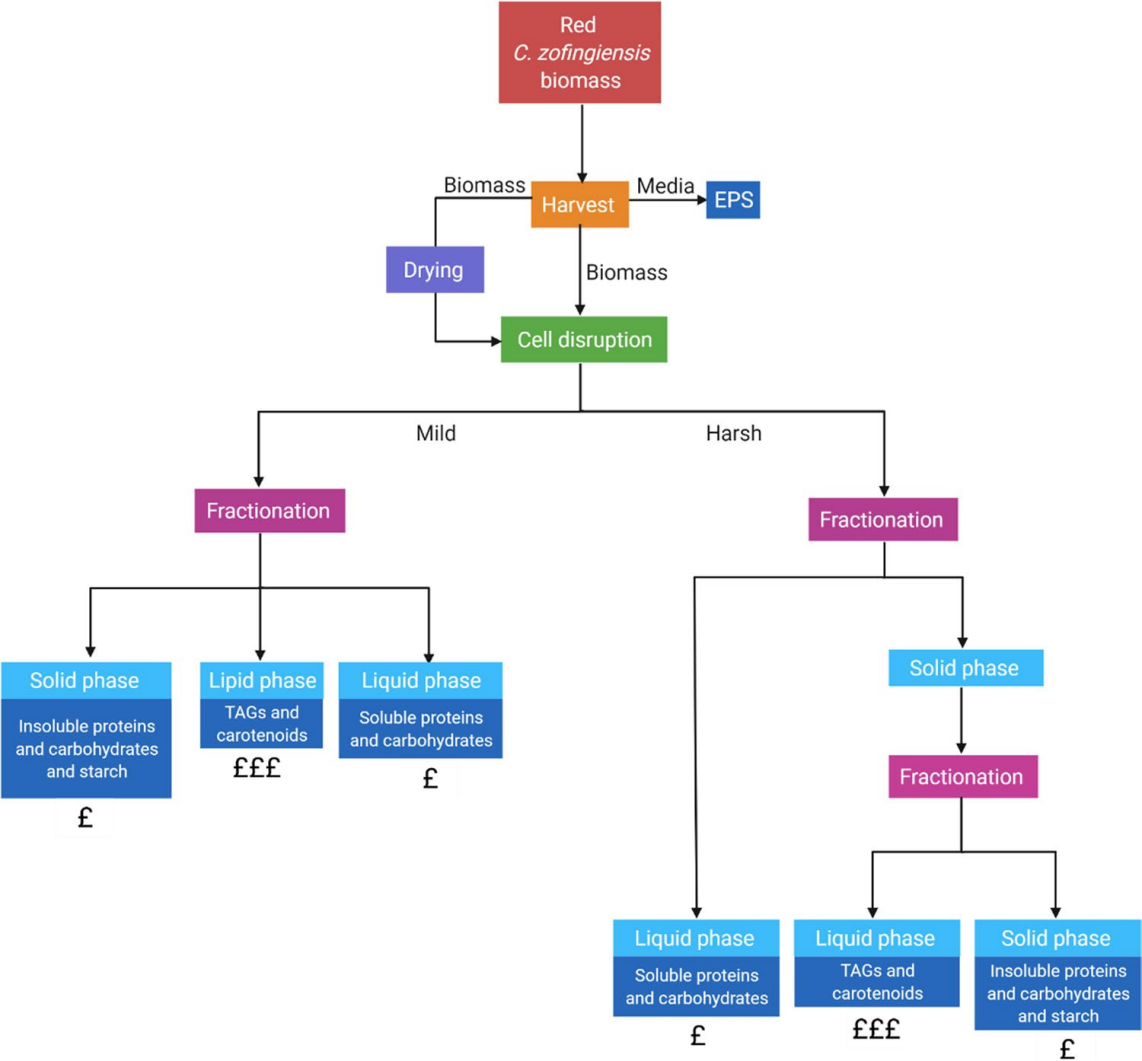
Theoretical biorefinery flowchart for obtaining multiple products from *C. zofingiensis* biomass. The dark blue boxes show the potential products from each stage of the process. The £ signs represent the market value of the individual products

There are many choices for the biorefinery configuration, due to the number of algal species investigated and products of principal interest; algal biorefineries are more of a “glass slipper” rather than a “one size fits all”. The species of microalgae and the specific strain are of extreme importance in the conceptual design, as they will determine the maximum biomass productivity and relative composition of the biomass constituents that will determine the most appropriate products for biorefinery [166]. The overall biorefinery process for *C. zofingiensis* could be successful via many different routes. The relationship between each process also needs to be fully understood so that the methods used at each stage can be optimised. The different options for cultivation, cell disruption, and separation of products are discussed throughout Sects. 4 and 5 and have been collated in Fig. 5. For any of these techniques to be feasible*,* experimentations into cell disruption and product separation need to advance and the separation of hydrophilic and hydrophobic components must be considered. The techniques used to disrupt the cells can be both harsh or mild depending on the optimisation of variables such as duration, number of cycles, and speed or power. Figure 6 shows the general routes that can be taken for biorefinery of this species where the methods in Fig. 5 can be applied. At the harvesting stage, there could be potential to obtain EPS which is freely associated in the culture medium depending on the other desirable products. Theoretically, if cell disruption is mild enough, solid, lipid, and liquid phases can be obtained once the cells have been broken. This is the model approach but has yet to be investigated. Alternatively, if harsh cell disruption is required, the lipids may remain in the solid phase with cell debris components or form an emulsion which will require further fractionation via solvent extraction. Once a lipid-carotenoid extract has been obtained astaxanthin could be further separated via solvent extraction.

## Challenges and future perspectives

**Fig. 7:**
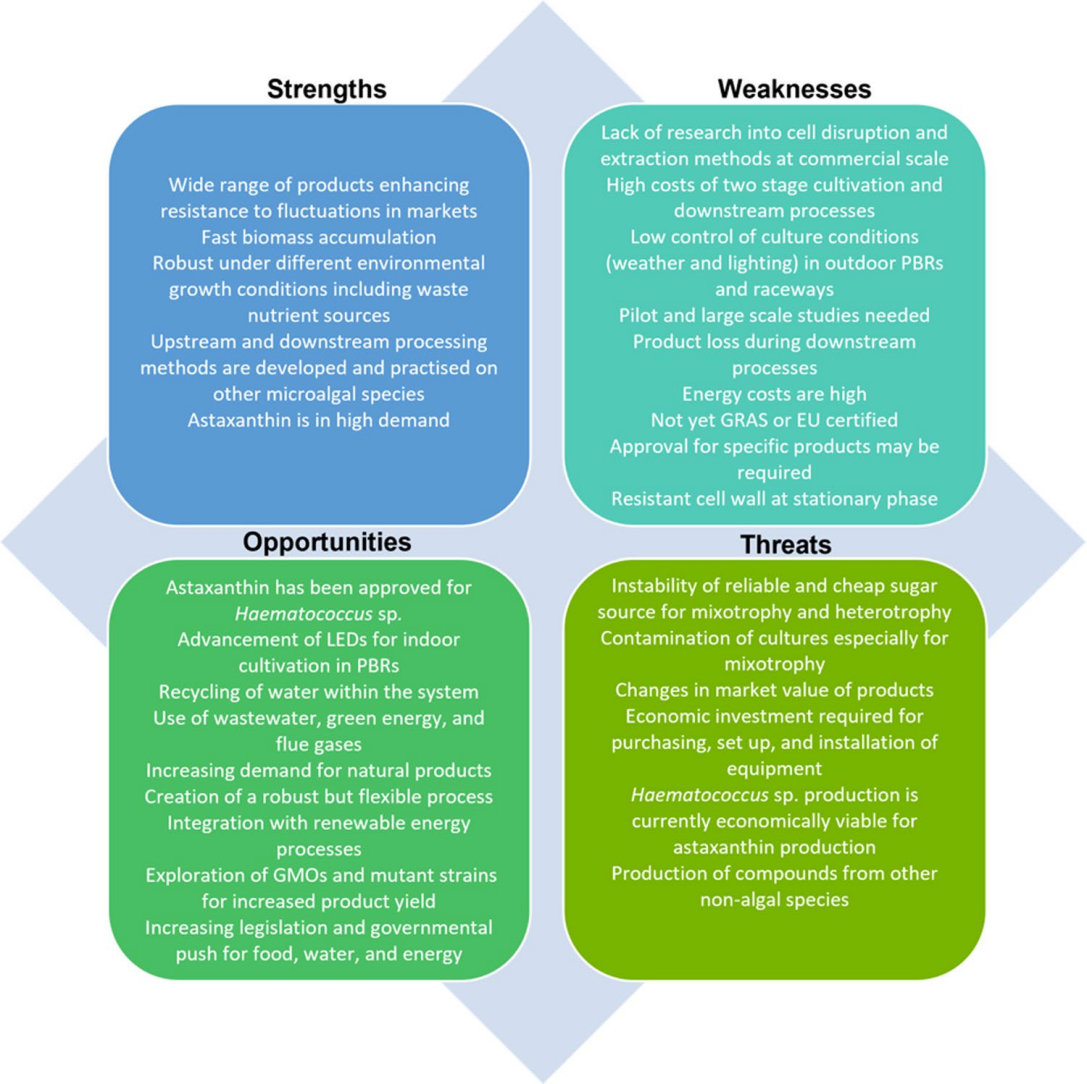
SWOT analysis for the potential for biorefinery of microalgae in general and in relation to *C. zofingiensis*

Developing biorefineries that are optimised and tailored for the production and processing of algal bioproducts is crucial [1]. In general, research needs to focus on advanced production, downstream, and bioprocess technologies to reduce costs. Efforts should also focus on the reduction of product loss and equipment and energy costs. Losses due to degradation also need to be addressed [185]. Once optimal techniques are established, they must be trialled in succession as the subsequent stage can be affected depending on the methods used. The algal pipeline needs to be researched thoroughly to establish a mechanism to achieve biorefinery of *C. zofingiensis.* This includes market opportunities, cultivation conditions, up- and downstream processing, demand, public perception, legislation, regulation, TEA, and LCA. The strengths, weaknesses, opportunities, and threats toward the potential of a *C. zofingiensis* biorefinery are presented in Fig. 7.

Recycling water within algal cultivation systems and the use of waste nutrient sources for biomass production could improve the economics of the astaxanthin-targeted biorefinery production of *C. zofingiensis* [9, 162]. *C. zofingiensis* can effectively bioremediate and grow on piggery, dairy, and municipal wastewater as well as anaerobic digestate [186–188]. There may also be opportunities to sequester or scrub flue gasses for carbon credits: where companies emitting CO_2_ can buy carbon capture by investing in green technologies to help abate environmental pollution and reduce operational costs [189, 190]. However, using certain types of waste can limit the potential markets that the end products can enter due to legislation as well as public perception. For example, growth on piggery wastewater can be undesirable socially so human food products or supplements may be unlikely to succeed. Although using wastewater may decrease the requirements of potable water and nutrient supplementation, it may not be possible to maintain stable process conditions since they are such dynamic matrices. For example, nutrient input is variable and there is a risk of heavy metal contamination. Moreover, contamination with bacteria and other microorganisms may be a major issue that could influence the purity of the products so waste or recycled water should be sterilised which can incur extra costs and energy input [166]. However, multi-purpose use of facilities such as membrane filtration can be employed here [127, 191]. Nevertheless, the use of recirculated water had no significant effect on the lipid content of *C. zofingiensis* implying that such a system could be applied for biofuel feedstock production [187]. It is however important to consider the stage at which the recycled water will be used and monitoring of nutrients and added sugars should take place. Zhu et al. [135] propose a biorefinery framework for *C. zofingiensis* grown in wastewater. The end products suggested are high-value products (fine chemicals, animal feed, human nutrition, protein, pharmaceuticals, cosmetics), biodiesel, biogas, and fertilisers. It is suggested that water, leftover nutrients, and CO_2_ from anaerobic digestion are recycled into the system for further biomass accumulation. However, the specific products that can be obtained from this species were not considered for this approach, nor was a stress stage incorporated into this framework, and the fractionation mechanisms were not identified. Therefore, the proposed biorefinery process may be limited in its true feasibility.

There is scope for integrating algal production with renewable energy systems. For example, the Algal Solutions for Local Energy (ASLEE) and Energy and the Bioeconomy (ENBIO) projects showed that algae can be grown using surplus local energy for PBR lighting to assist in grid balancing. Subhadra and Edwards (2010) propose an integrated Renewable Energy Park approach that combines different renewable energy industries, in resource-specific regions, for synergetic electricity and liquid biofuel production, enabling net-zero carbon emissions. A combination of wind power plant with solar panels and algal growth facilities could greatly optimise land for multi-stage product recovery. Biorefineries configured within these could produce biofuel, provide high-value co-products, and have almost zero environmental impact [162]. Liu et al. [9] specify the uses of the fractions from *C. zofingiensis* as astaxanthin, lipids for biofuel production, residual biomass after lipid extraction for nutraceuticals and animal feeds, and the carbohydrates for methane production by anaerobic digestion which could be used to power the plant. Such uses can assist in creating circular economies for more sustainable industrial approaches, although the land use required for biofuel production should be considered and non-arable land should be used [192].

There are also opportunities for the development of genetically modified organisms (GMOs) and mutant strains to improve quantities of desirable components. An example of a mutant is CZ-bkt1, created by chemical mutation and colour selection, which was generated to accumulate high quantities of zeaxanthin (7 mg/g) rather than astaxanthin [66]. This mutant also accumulated high concentrations of lutein (13.81 mg/g) and β-carotene (7.18 mg/g) [66]. The astaxanthin production economics of *C. zofingiensis* may be optimised by strain improvement via genetic engineering, development of next-generation culture systems, and the establishment of biorefinery production strategies [9]. The genome of this species has been sequenced which allows research of the molecular mechanism of astaxanthin biosynthesis which could in turn enhance its concentration [10, 138]. Nevertheless, the use of such organisms requires strict regulations which may limit the possible applications, i.e. use for human consumption, and there are often negative public opinions associated with GMOs.

The demand for natural products can be variable and the strength of biorefinery is that it offers flexibility for the manufacture of different products depending upon the demand. If a careful investigation into the conditions that cause the accumulation of specific products takes place, then the biorefinery approach will be more robust and resilient to changes in the market. As algal biotechnology advances, demand for such products may increase and public knowledge and acceptance will grow. When assessing biorefinery feasibility, one of the challenges is the lack of research on the economic performances and viability of processes at a commercial scale, especially in terms of astaxanthin production [193]. Techno-economic assessments (TEA) and life cycle assessments (LCA) are necessary when creating any new process or evaluating system performance [185]. Real-time projections for the prediction of cost per volume models are required for the extrapolation of lab-scale data and the development of marketable technologies [162]. Such analysis should consider how realistic the biorefinery process is, the commercial competition for example, with other microalgae, bacteria, and yeast, the demand, and the sustainability of the market values. To calculate the economic profitability, investment criteria are required as well as the prices of the inputs and outputs [194]. However, market values are continuously changing, and data is difficult and costly to obtain. Integration of unit operations also challenges the accuracy of TEA due to the complexity of the interactions between different processes.

TEA was performed in 2016 and 2020 for a range of species where it was concluded that microalgal biorefineries in general can be economically feasible [195, 196]. This is promising for the future of algal biorefineries; however, biorefinery costs vary significantly between species so TEA and LCA should be tailored carefully for the implementable and targeted biorefinery of *C. zofingiensis* [197]*.* From TEA on *D. salina* and *H. pluvialis*, it has been demonstrated that reactor type and location are of utmost importance due to varying climates, operational, and labour costs [194, 198, 199]. Both species have been described as suitable as part of an intermediate, rather than optimal, value chain when astaxanthin is one of the products, meaning that cost improvements cannot be made to one factor without deterioration of another [200]. This is due to the two-step process required which will incur increased capital and operational costs [198, 201, 202]. Table 5 displays the potential revenue from production of *C. zofingiensis* products under different growth modes. From this, heterotrophic growth could be the most promising for an economically viable biorefinery approach although these calculations have not considered the input costs: nutrients, carbon, energy, downstream processing, and labour, which will vary between growth modes. Additionally, costs will differ depending upon the scale as bulk buying may reduce input costs, although flooding the market should be avoided. Two studies were compared by Perez-Garcia et al. [199] which showed that the cost of biomass production under phototrophic conditions was higher than that in heterotrophic conditions ($5/kg compared to $1.4/kg, respectively) when estimated conservatively but once optimised, phototrophic was lower than heterotrophic ($0.5/kg versus $1.19/kg, respectively). If production costs are high, the process can only be economically feasible if products are sold as high-value commodities, for example, the use of lipids for nutraceuticals rather than for biodiesel [203].

**Table 5:** Estimated revenue of biorefinery products from *C. zofingiensis* under different growth conditions. Values were calculated based on the highest biomass concentration, highest quantity of product, and market prices in Table 1

Growth mode (red phase)	Highest biomass concentration (g/L)	Target product	Highest quantity of product (% DW)	Estimated revenue per 100,000L algal culture	References
Phototrophic	13.5	Astaxanthin Lipids Carbohydrates Proteins	0.03% 54.5% 66.9% 16.56%	$810–6075 $1103–58,826 $451–903,150 $111–8942 Total $2475–976,993	[10, 25, 83, 125, 141]
Mixotrophic	10.71	Astaxanthin Lipids Carbohydrates Proteins	0.24% 42.4% 37% 20%	$5140–38,556 $461–24,586 $198–396,270 $107–8568 Total $5906–467,980	[70, 101, 115]
Heterotrophic	98.4	Astaxanthin Lipids Carbohydrates Proteins	0.07% 53.1% 28% 16%	$13,776–103,320 $7837–418,003 $1377–2,755,000 $787–62,976 Total $23,772–3,339,299	[101, 109, 124, 149]
Heterotrophic-phototrophic	73.7	Astaxanthin Lipids Carbohydrates Proteins	0.27% n/a n/a n/a	$39,798–298,485 n/a n/a n/a	[61, 101]

## Conclusion

In conclusion, *C. zofingiensis* holds great potential for biorefinery and future commercialisation for production of astaxanthin, TAGs, carbohydrates, and proteins. The broad range of products makes this species a robust candidate for biorefinery as it will be adaptable to changes in market values. There are many different methods to induct the red phase of culture and the advantages and disadvantages of each must be considered prior to scale-up. However, the possibility of obtaining additional products besides astaxanthin and lipids needs to be investigated further. Analysis has identified many products which are suggested by-products, but these ideas need to be brought together to establish a specific integrated approach with consideration of current demand and market value. Harvesting via centrifugation or filtration must be investigated further to assist in obtaining a pure product because astaxanthin, the primary target compound, carries a high market value. Therefore, investing in a more expensive technology will be beneficial economically and contamination will be avoided. Cell disruption is a key area for further investigation to establish techniques that will enable careful fractionation of products to avoid unnecessary mixing and degradation. Using certain techniques in moderation or consecutively may be the best route to achieve this. Thorough research into fractionation processes is necessary as this area is the least explored for this species. Furthermore, the downstream process must be optimised and trialled in succession and then scaled. Despite many challenges and needs for development, if the correct research and investment are put in place this species could be one of the next commercialised natural producers of astaxanthin whilst simultaneously producing lipids specifically TAGs, carbohydrates such as starch or EPS, and proteins with a favourable amino acid content. If this can be achieved, such a biotechnological process will allow renewable production of multiple natural products and contribute towards a sustainable future.

### Supplementary Information

Below is the link to the electronic supplementary material.

Supplementary file 1 (DOCX 22.5 KB) 
