# Supplementary material for: Progress towards a targeted biorefinery of Chromochloris zofingiensis: a review
Source: Biomass Convers Biorefin. 2022 Jun 27;14(7):8127–52. doi: 10.1007/s13399-022-02955-7 (PMC10948469; doi:10.1007/s13399-022-02955-7)
Supplement: Supplementary file 1 — (DOCX 22.5 KB) [file 13399_2022_2955_MOESM1_ESM.docx]

**Supplementary information**

# Progress towards a targeted biorefinery of *Chromochloris zofingiensis*: a review.

Eleanor E. Wood^1,2^, Michael E. Ross^1^, Sébastien Jubeau^2^, Valéria Montalescot^3^, Michele S. Stanley^1^

^1^ University of the Highlands and Islands (UHI), Scottish Association for Marine Science (SAMS), Scottish Marine Institute, Oban PA37 1QA, UK

^2^ Xanthella Ltd, Malin House, European Marine Science Park, Dunstaffnage, Argyll, Oban PA37 1SZ, Scotland, UK

^3^ Cargill, 1 rue de Sèves, Baupte, France

Biomass Conversion and Biorefinery

*Corresponding author: Eleanor E Wood [eleanor.wood@sams.ac.uk](mailto:eleanor.wood@sams.ac.uk)

**Online Resource 1:** Strain of Chromochloris zofingiensis currently available from culture collections. Strain names filled with the same colour represent those that are from the same isolate.

| **Culture collection** | **Strain number** | **Culture collection name** | **Previous names and synonyms** | **Isolated** | **Same as** | **Reference** |
| --- | --- | --- | --- | --- | --- | --- |
| CCAP | 211/14 | *Chromochloris zofingiensis*. | Formerly (<2018) *Chlorella zofingiensis* Donz 1934. Synonyms: *Bracteacoccus cinnabarinus, Chlorella zofingiensis, Muriella zofingiensis, Mychonastes zofingiensis* | By Dönz 1933, Ramooswald near Zogingen, Switzerland. | Orig. desig. Zurich 205. ATCC 30412; IAM C-111; SAG 211-14; UTEX 32. | <https://www.ccap.ac.uk/strain_info.php?Strain_No=211/14> |
| CCAP | 221/2 | *Chromochloris zofingiensis* | Formerly (<2018) *Bracteacoccus cinnabarinus* (Kol & Chodat) Starr 1955. Synonyms: *Bracteacoccus cinnabarinus, Chlorella zofingiensis.* | Chodat, Switzerland | Orig. desig. V.162; SAG 221-2; UTEX 56. | <https://www.ccap.ac.uk/strain_info.php?Strain_No=221/2> |
| SAG | 211-14 | *Chromochloris zofingiensis* (Dönz) Fucikova & Lewis. | *Muriella zofingiensis* (Dönz) Hindak | *Chlorella zofingiensis* Dönz 1933. Switzerland, soil near Ramooswald near Zofingen. |  | <http://sagdb.uni-goettingen.de/showstrains.php?allfields=zofingiensis&strain_number=&prev_name=&genus=&division=&species=&class=&search=Show+strains> |
| SAG | 221-2 | *Chromochloris zofingiensis* (Dönz) Fucikova & Lewis. | *Chromochloris cinnabarina* Kol et F. Chodat. | Switzerland, soil from Unterengadin 1934 E.col and F. Chodat |  | <http://sagdb.uni-goettingen.de/showstrains.php?allfields=zofingiensis&strain_number=&prev_name=&genus=&division=&species=&class=&search=Show+strains> |
|  |  |  | Authentic strain of *Bracteacoccus cinnabarinus* (Kol et F. Chodat) Starr |  |  |  |
| SAG | 31.8 | *Chromochloris zofingiensis* (Dönz) Fucikova & Lewis. | *Muriella zofingiensis* (Dönz) Hindak. | 1963, F. C. Czygan. |  | <http://sagdb.uni-goettingen.de/showstrains.php?allfields=zofingiensis&strain_number=&prev_name=&genus=&division=&species=&class=&search=Show+strains> |
|  |  |  | *Chlorella zofingiensis* (Dönz). | Ortenberg near Marburg/Lahn Germany. |  |  |
| SAG | 34.8 | *Chromochloris zofingiensis* (Dönz) Fucikova & Lewis. | *Muriella zofingiensis* (Dönz) Hindak. | Germany, Ortenberg near Marburg/Lahn, on bark of deciduous tree |  | <http://sagdb.uni-goettingen.de/showstrains.php?allfields=zofingiensis&strain_number=&prev_name=&genus=&division=&species=&class=&search=Show+strains> |
|  |  |  | *Chlorella zofingiensis* (Dönz). |  |  |  |
| SAG | 4.8 | *Chromochloris zofingiensis* (Dönz) Fucikova & Lewis. | *Muriella zofingiensis* (Dönz) Hindak. | M. B. Allen. Strain number by isolator was C-1.2.1 as C. vulgaris (strain Z. 2.4.1 of Hopkins Mar. Stat). possibly 1952 if not earlier. |  | <http://sagdb.uni-goettingen.de/showstrains.php?allfields=zofingiensis&strain_number=&prev_name=&genus=&division=&species=&class=&search=Show+strains> |
|  |  |  | *Chlorella zofingiensis* (Dönz). *Chlorella* sp. |  |  |  |
| CAUP | H6006 | *Chromochloris zofingiensis* (Dönz) Fucikova & Lewis. |  | Hindak 1969 |  | <https://botany.natur.cuni.cz/algo/caup-list.html#Chlorophyceae> |
| CAUP | H6503 (not used in the literature) | *Chromochloris zofingiensis* (Dönz) Fucikova & Lewis. |  | Donz 1933 | CCAP 211/14, | <https://botany.natur.cuni.cz/algo/caup-list.html#Chlorophyceae> |
|  |  |  |  |  | SAG 211/14, |  |
|  |  |  |  |  | UTEX 32, |  |
|  |  |  |  |  | ATCC 30412, |  |
|  |  |  |  |  | IAM C-111 |  |
| CAUP | H6504 (not used in the literature) | *Chromochloris zofingiensis* (Dönz) Fucikova & Lewis. |  | Allen No, Z 2.4.1 | SAG 4.80 | <https://botany.natur.cuni.cz/algo/caup-list.html#Chlorophyceae> |
| MZCH | 10156 (not used in the literature) | *Chromochloris zofingiensis* | (class Trebouxiophyceae) |  |  | <http://www.mzch-svck.uni-hamburg.de/?action=details&strain_num=10156> |
| ATCC | 30412 | *Chlorella zofingiensis* Dönz |  | Switzerland 1933 | UTEX 32 [CCAP-211/14, Meyer No. 30] | <https://www.lgcstandards-atcc.org/products/all/30412.aspx?geo_country=gb#generalinformation> |
| CCALA | 944 | *Chlorella zofingiensis* Dönz |  | Italy, Firenze, isolator = Boussiba |  | <https://ccala.butbn.cas.cz/en/chlorella-zofingiensis-donz> |
| UTEX | 32 | *Chlorella zofingiensis* | Formerly C*. zopfingiensis [sic] (Starr & Zeikus 1987); C. miniata (Shihira & Krauss 1965); Chlorella miniata; Mychonastes zofingiensis* | O. C. Donz 1933, Ramooswald, Zopfingen [sic], Switzerland | Relatives: CCAP 211/14 aka Meyer 30; | <https://utex.org/products/utex-0032?variant=30991239348314> |
|  |  |  |  |  | SAG B 211-14; |  |
|  |  |  |  |  | ATCC 30412; |  |
|  |  |  |  |  | IAM C-111; |  |
|  |  |  |  |  | CAUP H 6503 |  |
| ACOI | 531 (not used in the literature) | *Chlorella zofingiensis* Dönz |  | 1990, Coimbra, Carvalho & Santos |  | <http://acoi.ci.uc.pt/spec_detail.php?cult_id=449> |
| UTEX | 56 | *Bracteacoccus cinnabarinus* | *Dictyococcus cinnabarinus* | Chodat, Switzerland | Relatives SAG 221-2; CCAP 221/2 [dec.] 8/51 to CCAP from Vischer 162; 1955 renamed (Starr 1955c). | <https://utex.org/products/utex-0056?variant=30991944548442> |
|  |  |  | *Chromochloris cinnabarina, Chromochloris zofingiensis* (Fucikova and Lewis, 2012)*.* |  |  |  |
| ACOI | 821 (not used in the literature) | *Bracteacoccus minutus* Schwarz |  | 1995 Leiria, Unknown isolator, identified by Santos |  | <http://acoi.ci.uc.pt/spec_detail.php?cult_id=169> |
| ASIB | S223 | *Bracteacoccus minutus* Schwarz |  | Schwarz 1979, Dalmatia, Croatia. |  | [1] |
| NIES | 2175 (not used in the literature) | *Muriella zofingiensis* (Dönz) Hindak | Synonym: *Chlorella zofingiensi*s Dönz | Ramooswald near Zofingen Switzerland. Dönz, O. C. (1933) |  | <https://mcc.nies.go.jp/strainList.do?strainId=2567&strainNumberEn=NIES-2175&condition=zofingiensis> |
| EGEMACC | 20 | *Chlorella zofingiensis* |  | Müge İşleten Hoşoğlu and Zeliha Demirel |  | http://www.egemacc.com/en/cultures.php |
| CCALA | 888 | *Mychonastes zofingiensis* | Synonym: *Chlorella zofingiensis* Donz | Hindak, 2009, Slovakia Sklene Teplice, thermal spring. | HINDAK /H 6007 CAUP | https://ccala.butbn.cas.cz/en/mychonastes-zofingiensis-doenz-t-kalina-et-m-puncocharova |
| CALU | 190 | *Chromochloris zofingiensis* |  |  | CCAP 211/14, UTEX 32, SAG 211/14, ATCC 30412. | [2]  Collection of Algae of Leningrad University. |

1. Fucikova K, Lewis LA (2012) Intersection of Chlorella, Muriella and Bracteacoccus: Resurrecting the genus. Fottea 12:83–93.

2. Minyuk G, Sidorov R, Solovchenko A (2020) Effect of nitrogen source on the growth, lipid, and valuable carotenoid production in the green microalga Chromochloris zofingiensis. Journal of Applied Phycology 1–13. doi: https://doi.org/10.1007/s10811-020-02060-0
